# Meandering Main Pancreatic Duct: A Rare Cause of Idiopathic Recurrent Acute Pancreatitis

**DOI:** 10.7759/cureus.87528

**Published:** 2025-07-08

**Authors:** Hara Prasad Mohapatra, Pradosh Kumar Sarangi, Nishit Ranjan, Amardeep Kumar

**Affiliations:** 1 General Surgery, All India Institute of Medical Sciences, Deoghar, IND; 2 Radiodiagnosis, All India Institute of Medical Sciences, Deoghar, IND

**Keywords:** idiopathic acute pancreatitis, magnetic resonance cholangiopancreatography (mrcp), meandering main pancreatic duct, n-shaped meandering main pancreatic duct, recurrent acute pancreatitis

## Abstract

The meandering main pancreatic duct (MMPD) is a rare developmental variant associated with idiopathic recurrent acute pancreatitis (IRAP) due to potential ductal hypertension. Although acute evaluation by magnetic resonance cholangiopancreatography (MRCP) is not routinely recommended for acute pancreatitis, it becomes essential in unexplained cases. We report a case of a 19-year-old male who presented with acute epigastric pain and recurrent pancreatitis, marked by elevated serum lipase and a bulky pancreas on CT. MRCP revealed an MMPD with a unique N-shape, without ductal dilatation or intraductal calcification and gallstones or other identifiable causes, suggesting it as the etiology of his IRAP. He was managed conservatively, with resolution of symptoms, and advised to avoid potential risk factors and report any recurrence. Some cases of recurrent acute pancreatitis have no identifiable cause, but anatomical variations of the pancreatic duct, such as an MMPD, are thought to play a contributory role, although the underlying mechanism is not fully understood. Early recognition of such anomalies through imaging, such as MRCP, is important to identify the underlying causes of IRAP and guide appropriate long-term management.

## Introduction

Acute pancreatitis is a serious condition that can have life-threatening consequences. Its common causes include alcohol use, gallstones, autoimmune conditions, trauma, and anatomical abnormalities such as anomalous pancreaticobiliary junction and pancreatic divisum [1-4]. Approximately 20% of pancreatitis cases are classified as idiopathic, with a 30% recurrence rate [4]. Recurrent episodes are frequently linked to dilation of the pancreatic duct.

Anatomical variants of the pancreatic ductal system are recognized contributors to acute and recurrent acute pancreatitis (RAP). These include anomalous pancreaticobiliary junction, pancreas divisum, annular pancreas, ansa pancreatica, and the meandering main pancreatic duct (MMPD). Such anomalies can impair pancreatic drainage, predisposing individuals to ductal hypertension and inflammation [1-5]. MMPD was the contributing factor in 40% of patients with idiopathic pancreatitis according to one study [4].

Typically, the main pancreatic duct (MPD) follows an obtuse angle, as it extends from the tail and body of the pancreas to the major papilla. However, in some cases, the ventral pancreatic duct in the head region may exhibit an abnormal curvature, forming a localized spiral or hairpin shape, an anomaly known as the meandering pancreatic duct. This abnormality can lead to ductal hypertension and may contribute to episodes of idiopathic recurrent pancreatitis [1,4-6].

While magnetic resonance cholangiopancreatography (MRCP) is not usually performed during the acute phase of pancreatitis, it is valuable for assessing patients with unexplained or recurrent pancreatitis, as it provides a non-invasive, detailed visualization of the biliary and pancreatic duct systems. The meandering pancreatic duct, a rare anatomical variant, can be a potential cause of idiopathic recurrent acute pancreatitis (IRAP) [4,6]. This condition is typically diagnosed through endoscopic retrograde cholangiopancreatography or MRCP [4-7].

In this report, we describe a case of acute recurrent pancreatitis associated with a unique N-shaped meandering pancreatic duct.

## Case presentation

A 19-year-old male presented with acute epigastric pain persisting for two days, characterized by sharp, constant pain radiating to the back, exacerbated by food intake, alleviated by analgesics, and relieved by leaning forward. The episodes were accompanied by four to five bouts of non-bilious vomiting, without any associated fever or shortness of breath. There was no history of hematemesis, hemoptysis, bleeding per rectum, melena, or jaundice. Similar episodes occurred twice in the past, two years ago and one year ago, both of which were managed conservatively with IV fluids and analgesics during hospitalization. He has no known medical or surgical history and consumes a typical home-cooked Indian diet, including both vegetarian and non-vegetarian foods, with occasional alcohol intake (one to two times, every six months). Family history is unremarkable for pancreatitis. Systemic evaluation was unremarkable.

On clinical evaluation, the patient was alert, well-oriented, and of moderate build. Vital signs were within normal limits, with a temperature of 97.5°F, a pulse rate of 86 beats per minute, a blood pressure of 116/76 mmHg, a respiratory rate of 16 breaths per minute, and a body weight of 59 kg. There were no clinical signs of pallor, icterus, or jaundice. Abdominal examination revealed mild epigastric tenderness, soft abdomen, without organomegaly, palpable lump, or shifting dullness, with normal bowel sounds. The laboratory findings revealed significantly elevated serum lipase levels, while serum amylase was mildly above the reference range, supporting a diagnosis of pancreatic involvement. Liver function tests revealed abnormalities, including raised total and direct bilirubin levels, with mildly elevated alkaline phosphatase. However, transaminases were within normal limits. Additionally, leukocytosis was present with neutrophilia, indicating a possible underlying inflammatory response. Other parameters, including kidney function test, serum calcium, lipid profile, and serum lactate dehydrogenase, were within normal limits (Table 1). Ultrasonography revealed a mildly bulky and inflamed pancreas with a few peripancreatic lymph nodes, a normal gall bladder, and a normal diameter of the common bile duct (CBD), without any evidence of choledocholithiasis or mass lesion. Contrast-enhanced CT of the abdomen revealed a bulky body and tail of the pancreas with a normal MPD measuring 2.5 mm, without evidence of calculi or collections around and in the pancreatic tissue.

**Table 1:** Laboratory parameters observed in the patient and their normal ranges. HDL: high-density lipoprotein; LDL: low-density lipoprotein; VLDL: very low-density lipoprotein; TLC: total leukocyte count

Laboratory parameter	Observed values	Normal range
Serum lipase (U/L)	443.1	0–160
Serum amylase (U/L)	90	23–85
Total bilirubin (mg/dL)	1.57	0.2–1.2
Direct bilirubin (mg/dL)	1.26	0.1–0.3
Indirect bilirubin (mg/dL)	1.31	0.1–0.9
Alkaline phosphatase (U/L)	152	44–147
Alanine aminotransferase (U/L)	18	0–31
Aspartate aminotransferase (U/L)	26	5–34
Total protein (g/dL)	7.76	6.5–8.3
Albumin (g/dL)	4.78	3.5–5.0
Globulin (g/dL)	3.0	2.6–4.6
Albumin/globulin ratio	1.6	1.5–2.5
C-reactive protein (mg/L)	31.4	3.0–10.0
Blood urea nitrogen (mg/dL)	11.59	5.0–23.0
Blood urea (mg/dL)	25.28	10–45
Serum creatinine (mg/dL)	0.84	0.2–1.2
Serum sodium (mmol/L)	137	136–145
Serum potassium (mmol/L)	4.0	3.5–5.5
Serum chloride (mmol/L)	99	96–108
Serum calcium total (mg/dL)	9.68	8–10.2
Total cholesterol (mg/dL)	83.18	<200
Triglycerides (mg/dL)	101.89	<150
HDL cholesterol (mg/dL)	13.40	>40
LDL cholesterol (mg/dL)	81.57	<100
VLDL cholesterol (mg/dL)	20	2–30
Hemoglobin (g/dL)	14.7	12.6–17.1
TLC (×10³/μL)	14.68	4–11
Polymorphs (%)	76.5	40–75
Lymphocytes (%)	10.4	20–40
Monocytes (%)	7.3	2–10
Eosinophils (%)	5.8	0–6
Basophils (%)	0.0	0–2
Absolute neutrophil count (10³/μL)	11.22	2.0–7.0
Absolute lymphocyte count (10³/μL)	1.53	0.8–4.0
Absolute eosinophil count (10³/μL)	0.85	0.02–0.50
Absolute monocyte count (10³/μL)	1.08	0.12–1.20
Absolute basophil count (10³/μL)	0.00	0–2
Platelet count (×10³/μL)	154	150–400

**Figure 1:**
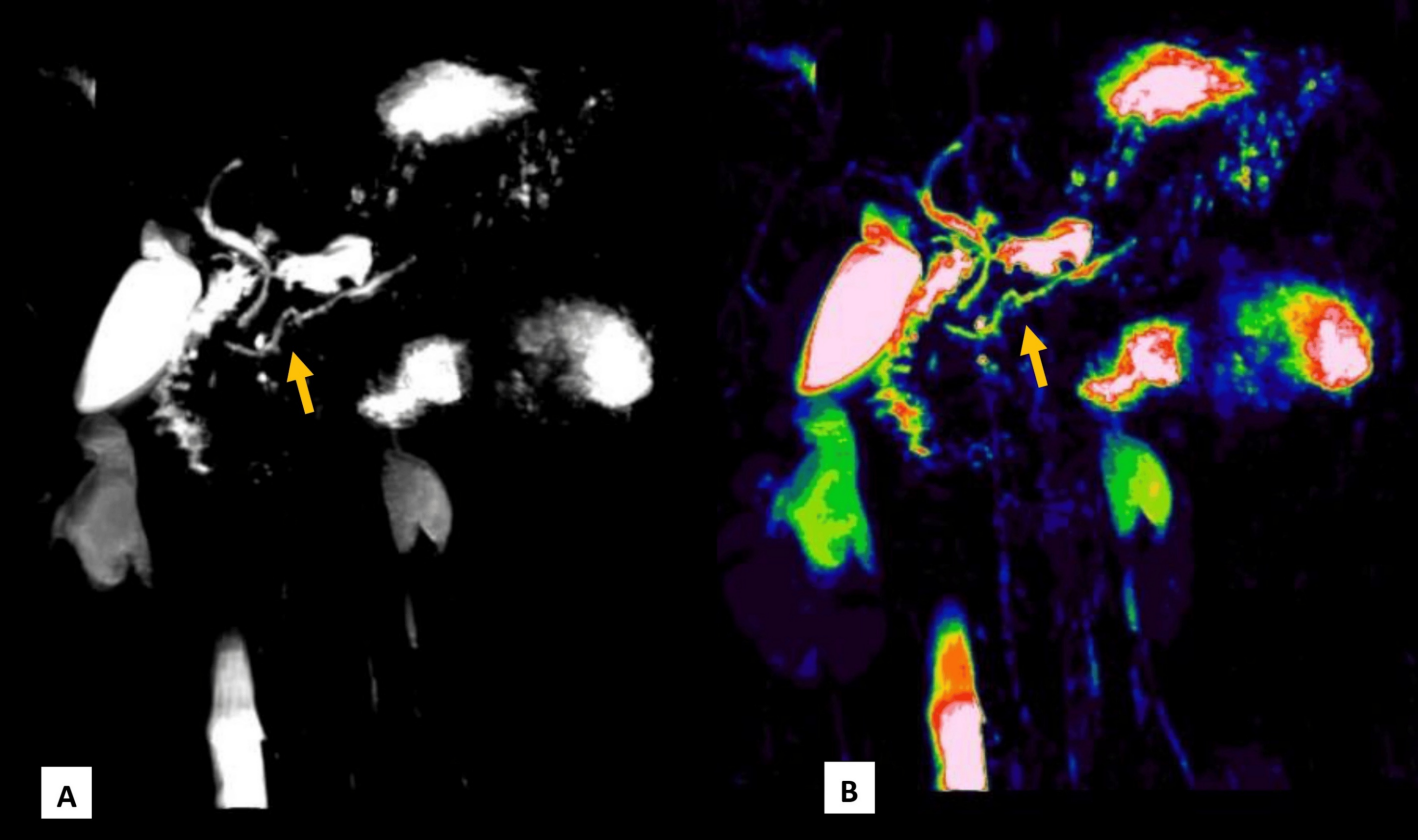
Coronal MRCP image (A) demonstrates an N-shaped MMPD in the head region of the pancreas. Image (B), processed with a color lookup table, enhances visualization of the MPD anatomy for better delineation. MMPD: meandering main pancreatic duct; MPD: main pancreatic duct; MRCP: magnetic resonance cholangiopancreatography

**Figure 2:**
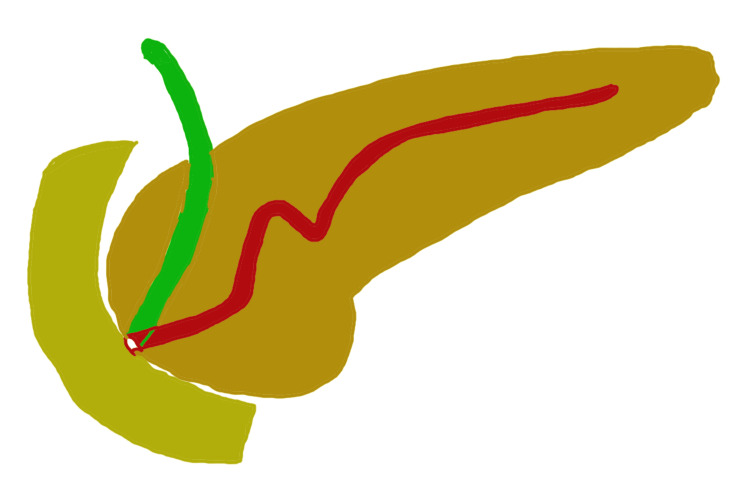
Schematic illustration depicting the N-shaped configuration of the MMPD. MMPD: meandering main pancreatic duct The figure was co-created by Dr. Pradosh Kumar Sarangi, Assistant Professor, Department of Radiodiagnosis and Dr. Himel Mondal, Department of Physiology, All India Institute of Medical Sciences (AIIMS), Deoghar.

As common causes of acute pancreatitis, including gallstones, alcohol use, hypertriglyceridemia, and drug-induced pancreatitis, were ruled out, further evaluation with MRCP was planned to look for CBD stones and to assess for ductal anomalies as a possible underlying etiology. It showed a bulky distal body and tail region of the pancreas consistent with acute pancreatitis, with no peripancreatic collections or pseudocysts. The MPD was normal in size but showed a unique N-shape in the pancreatic head region, suggestive of a MMPD (Figures 1A, 1B). There was no evidence of cholelithiasis or choledocholithiasis. Figure 2 shows a schematic illustration of the N-shaped configuration of the MMPD. 

The patient was managed conservatively with symptomatic treatment for acute pancreatitis. Supportive care included IV fluids, analgesics, and bowel rest. Over the course of a two-week hospitalization, clinical symptoms gradually improved, with resolution of abdominal pain. Laboratory parameters, including serum lipase and inflammatory markers, returned to baseline levels. The patient showed steady recovery and was discharged in a stable condition, with advice to avoid potential risk factors and to promptly report to the hospital in case of any similar future episodes. At six-month follow-up, the patient remained asymptomatic, with no recurrence of symptoms.

## Discussion

The root cause of RAP warrants investigation, as the condition can recur and may progress to chronic pancreatitis. Approximately 20% to 30% of recurrent cases are classified as idiopathic [1,2]. MRCP is a non-invasive imaging technique that effectively visualizes pancreatic duct anatomy and can reveal anatomical variations. One such uncommon variation is the MMPD, which typically shows an abnormal configuration in the head region of the pancreas.

Gonoi et al. [4] identified two distinct types of meandering MPD: the loop type and the reverse Z-type. This anomaly may contribute to recurrent episodes of acute pancreatitis. A cross-sectional study conducted at a tertiary referral center in Japan reported a 2.2% prevalence of meandering MPD in the general population, with a significantly higher occurrence of 40% among patients with RAP [4].

In a recent retrospective cross-sectional study of 214 patients in Finland, Johansson et al. [7] identified a unique MPD morphology known as the N-type, in which the MPD forms a deep or small notch in the head of the pancreas, where the duct of Santorini or the ansa pancreatica may unite within that notch. The N-type configuration was observed in 2% of healthy individuals. In patients with intraductal papillary mucinous neoplasm, this N-shaped duct morphology was found to be positively associated with the presence of cystic mural nodules, suggesting it may serve as a potential risk indicator and warrant closer follow-up in such cases [8]. This rare ductal morphology is infrequently described in the literature.

Although its precise etiology remains uncertain, MMPD is thought to resemble developmental variations of the pancreatic structure. The exact mechanism by which this abnormality induces pancreatitis is not fully understood. In our case, we have ruled out other causes behind recurrent pancreatitis. Previous literature has described loop-type and reverse Z-type configurations of the MMPD [4,9-11]. In our case, MRCP revealed an N-shaped MPD, a variant that has been recently reported. 

RAP is typically characterized by two or more clearly documented episodes of acute pancreatitis, with complete resolution of symptoms between episodes and no imaging or clinical evidence of chronic pancreatitis [9]. The present case met these diagnostic criteria. RAP is most commonly associated with alcohol consumption or gallstones. However, in approximately 10% to 30% of cases, the underlying cause remains unidentified during the initial workup, leading to a diagnosis of IRAP [4,9-12].

## Conclusions

The MMPD stands out as an exceedingly uncommon and rare pancreatic ductal anomaly, predominantly observed in patients grappling with acute recurrent pancreatitis. Before conclusively attributing it as the cause of idiopathic pancreatitis, it is imperative to eliminate other potential causes meticulously. Familiarity with diagnosing these lesser-known anatomical variations, especially N-shaped MMPD, becomes pivotal, as they may serve as predisposing factors for IRAP.
